# A Case of Poisoning with Sulphate of Copper

**Published:** 1870-01

**Authors:** D. B. Hillis

**Affiliations:** Keokuk, Iowa


Article VII.—*A Case of Poisoning with Sulphate of Copper.* Reported by Dr. D. B. Hillis, Keokuk, Iowa.

At 11 o'clock, on the night of the fifth of August, 1869, I was called to see Miss W. R., a large and well developed servant girl, æt. 18, doing housework in the family of Mr. O. of this city.

I was informed by him that the girl was in good health until about dusk of that evening, when, "all at once," she complained of intense pain in the head, soon followed by convulsions, and cramps in the stomach and bowels. When I arrived, she was having severe clonic spasms of the jaws, arms and legs; and, when able to speak, complained bitterly of the pains, especially of those of the stomach, declaring that she was "burning up." The pulse was full, hard, and 120 per minute. Anything like continued pressure on the epigastric region caused great suffering.

I readily concluded the case to be one of poisoning, by some agent of great virulence, which she, after some persuasion, admitted, but obstinately refused to say what it was, or where obtained. She coveted death; and positively refused to swallow anything, until I threatened to use the stomach pump, which I made her believe was an engine of great power. I gave her the whites of six eggs, all that could be had in the house, followed by copious draughts of sweet milk, and, in a short time, by an emetic of ipecac and tartarized antimony, which acted promptly, causing the ejection of a large amount of ingesta, intermixed with a dark-brown fluid.

To enforce this treatment, required at least one hour and a half from the time I first saw her. The spasms, of which she had not less than thirty in my presence, began to decrease, and subsided entirely within the next hour. I was of the opinion that she had taken arsenic, and, after giving the last emetic, had sent for the hydrated sesquioxide of iron; but before the messenger returned with it, the patient, having concluded that she could not die, as was her desire, and being exceedingly anxious for relief from the agonizing pains, admitted that, about two o'clock that afternoon, she had taken two spoonsfull of blue vitriol, in brandy; but whether large or small spoons, solid or fluid measure, she would not tell. The true enemy now being known, I changed front accordingly, and gave the prussiate of potassa, in fifteen grain doses, every hour, until the active symptoms of poisoning ceased. With the second portion of the antidote, a large dose of castor oil was given, for the purpose of removing from the intestines any copper they might contain.

Suffice it to say, that after the third portion of the potassa — about twelve hours after the poison had been taken — I left my patient enjoying a quiet sleep, and from that time on there were no more active indications of the presence of the agent which had given so much trouble, save the severe corrosion, or inflammation of the membranes, with which it had come in contact, and from which she recovered, under the use of aconite and belladonna, in tincture, together with demulcent drinks, and hot woolen cloths to the surface. I will just add, in conclusion, that for me the case was possessed of three points of special interest. The first, the length of time, five hours intervening, from the taking the poison until the development of the first symptom; the second, the entire absence of the usual nausea and vomiting produced by the various preparations of copper; and the third was, the efficiency and reliability, of the prussiate of potassæ as an antidote in such cases.

